# Fenretinide (4-HPR) Targets Caspase-9, ERK 1/2 and the Wnt3a/β-Catenin Pathway in Medulloblastoma Cells and Medulloblastoma Cell Spheroids

**DOI:** 10.1371/journal.pone.0154111

**Published:** 2016-07-01

**Authors:** Barbara Bassani, Desirèe Bartolini, Arianna Pagani, Elisa Principi, Massimo Zollo, Douglas M. Noonan, Adriana Albini, Antonino Bruno

**Affiliations:** 1 Scientific and Technological Pole, IRCCS MultiMedica, Milano, Italy; 2 Department of Molecular Medicine and Medical Biotechnology, University of Naples Federico II, Naples, Italy; 3 Ceinge Biotecnologie Avanzate, Naples, Italy; 4 Department of Biotechnology and Life Sciences, University of Insubria, Varese, Italy; University of Navarra, SPAIN

## Abstract

Medulloblastoma (MB), a neuroectodermal tumor arising in the cerebellum, represents the most frequent childhood brain malignancy. Current treatments for MB combine radiation and chemotherapy and are often associated with relevant side effects; novel therapeutic strategies are urgently needed. N-(4-Hydroxyphenyl) retinamide (4-HPR, fenretinide), a synthetic analogue of all-trans retinoic acid, has emerged as a promising and well-tolerated cancer chemopreventive and chemotherapeutic agent for various neoplasms, from breast cancer to neuroblastoma. Here we investigated the effects of 4-HPR on MB cell lines and identified the mechanism of action for a potential use in therapy of MB. Flow cytometry analysis was performed to evaluate 4-HPR induction of apoptosis and oxygen reactive species (ROS) production, as well as cell cycle effects. Functional analysis to determine 4-HPR ability to interfere with MB cell migration and invasion were performed. Western Blot analysis were used to investigate the crucial molecules involved in selected signaling pathways associated with apoptosis (caspase-9 and PARP-1), cell survival (ERK 1/2) and tumor progression (Wnt3a and β-catenin). We show that 4-HPR induces caspase 9-dependent cell death in DAOY and ONS-76 cells, associated with increased ROS generation, suggesting that free radical intermediates might be directly involved. We observed 4-HPR induction of cell cycle arrest in G1/S phase, inactivated β-catenin, and inhibition of MB cell migration and invasion. We also evaluated the ability of 4-HPR to target MB cancer-stem/cancer-initiating cells, using an MB spheroids model, followed by flow cytometry and quantitative real-time PCR. 4-HPR treatment reduced DAOY and ONS-76 spheroid formation, in term of number and size. Decreased expression of the surface markers CD133^+^ and ABCG2^+^ as well as *Oct-4* and *Sox-2* gene expression were observed on BTICs treated with 4-HPR further reducing BITIC invasive activities. Finally, we analyzed 4-HPR ability to inhibit MB tumor cell growth *in vivo* in nude mice. Taken together, our data suggest that 4-HPR targets both parental and MB tumor stem/initiating cell-like populations. Since 4-HPR exerts low toxicity, it could represent a valid compound in the treatment of human MB.

## Introduction

Medulloblastoma (MB) is a highly aggressive pediatric tumor of the cerebellum, usually located in the *posterior fossa* and represents the most common malignancy of the cerebellum in childhood, accounting for 13–20% of all pediatric central nervous system tumors [1, 2].

Current treatments include the combination of surgical resection, whole brain and spinal cord radiation and aggressive systemic multidrug-chemotherapy [3, 4]. These combined approaches have significantly boosted 5-year survival rates beyond 80%, [5] improving patient survival, however, these aggressively treated children can develop serious long-term side effects [6, 7].

Recently, different molecular subtypes of MB have been identified, on the basis of gene expression and immunohistochemistry differences and have been described as Wingless (Wnt), Sonic Hedgehog (SHH), Group 3 and Group 4 [1, 4, 8–12]. This knowledge has also strongly influenced the clinical therapy and possible intervention strategies, allowing a deeper understanding of the different mechanisms involved in MB genesis and development and in responsiveness to chemotherapy [11, 13].

The Wnt molecular subtype correlates with a good prognosis [14], Group 3 MB were associated with a worse outcome, while SHH and Group 4 patients displayed an intermediate prognosis [1, 4, 8–12]. The knowledge of the MB molecular profiling has led to several attempts at targeted therapies [14, 15] in preclinical studies and still open clinical trials that focused their attention mainly on SHH pathway antagonists, and among all the inhibitors of Smoothened (SMO) [11, 13]. However, mostly of these molecules might be ineffective in a clinical context due to secondary resistence onset in treated patients, suggesting that further studies are needed [12, 13].

The synthetic retinoid N-(4-hydroxyphenyl)retinamide (4-HPR, or fenretinide), a cancer chemopreventive and therapeutic agent [16–19] showed enhanced activity and reduced toxicity compared to natural retinoids *in vitro* and in clinical studies. 4-HPR is able to induce biological effects and apoptosis in several cancer cell lines [20], in particular in breast cancer cells [17, 21–23], prostate carcinoma cells [24–26], human pancreatic cancer cells [27] and myeloid leukemia [28]. Moreover, 4-HPR has been employed successfully in several clinical trials for the treatment of breast [17, 29–31] and prostate cancer [25]. Among the mechanisms by which 4-HPR induces cell death, the enhancement of reactive oxygen species (ROS) production and mitochondrial damages have been shown to play a crucial role [32, 33]. 4-HPR has proven to be effective also on cells of neuroectodermal origin such as neuroblastoma [34–36], gliomas [14, 37, 38] and melanoma [39]. Preliminary evidence of action on MB cells is available [18]. 4-HPR has been reported to affect the survival and to induce cell death in MB cell lines, modulating caspase-3 activation and PARP-1 cleavage [18].

Experimental and studies indicate the ability of 4-HPR to cross the blood brain barrier (BBB), indicating its potential in the treatment of brain pathologies [37, 38, 40].

In the context of MB heterogeneity [41], a cell population was identified from primary MB that displayed marked ability of proliferation, self-renewal and differentiation, demonstrating the presence of cancer stem like/initiating cells (CSCs/CICs), termed MB brain tumor-initiating cells (BTICs) [42]. CSCs/CICs showed several characteristics including poor differentiation, low rates of replication and expression of drug-resistance pumps that are associated with chemoinsensitivity [43–46]. The concept of CSCs came from the experimental evidence that only a small subpopulation of tumor cells is able to give rise to tumors *in vivo* [43, 44]. CSCs, when transplanted in an *in vivo* model, give rise to a tumor masses that recapitulate all the features of the original tumor [43, 44]. CSCs, due to their slow replication rates and expression of drug-resistance pumps, escape standard chemotherapeutic treatments, thus they are clearly implicated in tumor relapse and metastatic disease [45, 47]. Like the stem cells of normal tissues, CSCs are thought to undergo asymmetric division [43, 46, 48, 49]. BTICs represent a significant target for MB therapy due to their relevance in relapse and metastatic disease [50–52].

Culturing cells in spheroids has been considered a valid model for the isolation and investigation of CIC-CSC like tumor cells [41, 43, 44, 53].

Here, we evaluated the effects of 4-HPR on two widely used and characterized human MB cell lines, DAOY and ONS-76. We found that 4-HPR induces caspase 9-dependent cell death in DAOY and ONS-76 cells, as far as ROS generation. 4-HPR was able to promote cell cycle arrest in DAOY cells. 4-HPR targets ERK 1/2 and the Wnt3a/β-catenin pathway and inhibits MB cell migration and invasion. In the spheroid model for brain tumor initiating cells (BTICs) induction, 4-HPR was able to inhibit, in terms of size and number, spheroid formation. We also observed decreased levels of CD133^+^ and ABCG2^+^ cells as well as *Oct-4* and *Sox-2* gene expression in 4-HPR treated BTICs and inhibited invasive properties. Finally, we analyzed 4-HPR ability to inhibit MB tumor cell growth *in vivo* in nude mice.

Altogether, our data demonstrated that 4-HPR is able to target both parental and MB tumor stem/initiating cell-like populations, suggesting that fenretinide might represent a valid compound in the treatment of human MB.

## Materials and Methods

### Cell Culture

Two different and well characterized cell lines were used in our study, DAOY cells that directly derived from primary MB and ONS-76, described as a more immature cell line with a primitive profile [54]. DAOY and ONS-76 human MB cell lines were purchased from the American Type Culture Collection, (Manassas, VA). DAOY cells were cultured in Eagle's minimal essential medium (EMEM) supplemented with 1% Penicillin/Streptomycin and 10% fetal bovine serum (FBS) (all from Sigma-Aldrich, Milan, Italy) while ONS-76 was cultured in RPMI 1640 (GIBCO Cell Culture, Burlington, Ontario) containing 10% FBS, 1% L-glutamine 2 mM and 1% Penicillin/Streptomycin (Sigma-Aldrich). Cells were treated with 4-HPR (a kind gift from Dr. James A. Crowell, Division of Cancer Prevention, National Cancer Institute, Bethesda, MD, and Dr. Gregg Bullard, McKessonBio, Rockville, MD) dissolved in absolute ethanol in a stock solution of 10 mM and used at indicated concentrations for different time periods.

### Cell Proliferation assays

The effect of 4-HPR on MB cell proliferation was determined by 3-(4,5-dimethythiazol-2-yl)-2,5-diphenyl tetrazolium bromide (MTT, Sigma Aldrich) assay. Briefly, 1 x10^3^ cells were seeded into 96-well plates and incubated at 37°C overnight. The medium was then replaced with fresh 4-HPR containing medium (range 100 nM- 10 μM) and incubated for 24, 48, 72 or 96 h. MTT reagent 5 (mg/ml) was added to cell cultures and incubated for 3 hours at 37°C. After incubation, medium was replaced with 100 μL of DMSO and the amount of solubilized formazan was quantified at 570 nm in a SpectraMax M2 (Molecular Devices, Sunnyvale CA).

### Determination of apoptotic effect by Flow Cytometry

DAOY and ONS-76 cells in logarithmic phase of growth were treated with 4-HPR (range 1 μM– 20 μM) for 6 and 24 h. At the end of the incubation, cells were collected and stained with FITC-conjugated Annexin V (Becton Dickinson (BD) Bioscences, San Jose, CA) and 7-AAD (BD) in Annexin V Buffer 1X (0,1 M HEPES pH 7,4, 1,4M NaCl, 25 mM CaCl_2_ and H_2_O). This method allows discrimination between viable cells (FITC negative, 7-AAD negative), early apoptotic cells with intact cell membranes (FITC positive), late apoptotic (FITC positive, 7-AAD positive), and dead cells (FITC negative, 7-AAD positive). Analysis was performed on 10,000 gated cells to exclude cell debris using a FACSCanto (BD), with excitation set at 488 nm and emission at 518 nm (FITC detector) and 620 nm (7-AAD fluorescence detector).

### Cell cycle analysis

Asyncronized DAOY and ONS-76 cells were treated with 4-HPR (5–10 μM) or Vincristine (50 μM) as positive control, for 24h trypsinized, and fixed in cold 70% ethanol. DNA was stained with 100 μg/ml propidium iodide (PI) (Sigma- Aldrich) in hypotonic citrate buffer with 20 μg/ml ribonuclease A. Stained nuclei were analyzed for DNA-PI fluorescence using a FACSCanto flow cytometer. Resulting DNA distributions G0/G1, S, G2/M and apoptotic phase of the cell cycle were analyzed with FACSDiva software. 4-HPR effects on cell cycle were also investigated by detection of phosphorylated ciclin-kinase2 (anti-phospho-Chk2, Cell Signaling Technology, Danvers, MA) levels by cytofluorimetric analysis. Cells treated with 10 μM Etoposide were used as positive control. Briefly, cells were detached, collected by centrifugation and fixed/permeabilized using Cytofix-Cytoperm solution (BD) for 10 minutes at 4°C at dark. Unconjungated phospho-Chk2 primary antibody was added to each tube for 1h at room temperature. Cells were then washed and incubated with the secondary antibody conjugated with Alexa Fluor 482 (Life Technologies, Monza, Italy) for 30 minutes at room temperature. Cells were then rinsed, resuspended in PBS 1X and analyzed by flow cytometry. Expression levels of Cyclin D1 and CDK4 were detected by Western Blot analysis (see below).

### Determination of cellular ROS

ONS-76 and DAOY at 5 x 10^5^ cells/ml were treated with 5 and 10 μM 4 HPR for 2h. Treatment with H_2_O_2_ 100 μM for 15min at 37°C was used as positive control. Cells were incubated with 50μM 2',7'-dichlorodihydrofluorescein diacetate (H_2_DCFDA, Sigma-Aldrich) 20 minutes before the end of the treatment, washed twice with PBS and resuspended in the same medium. Cells were then analyzed with a flow cytometer FACSCantoII (BD Biosciences, San Jose, CA, USA) with excitation set at 488 nm and emission at 530 nm. The analysis was performed on 10,000 gated cells. Pre-incubation with 10 mM N-Acetyl-Cysteine (NAC, Sigma-Aldrich) for 1h was performed to evaluate its scavenger activity on ROS production induced by 4-HPR.

### Spheroids formation and stemness assessment

DAOY and ONS-76 human MB cells were cultured as described until 70% confluence. Cells were then detached by trypsinization and spheroid formation was performed by plating 1 x 10^4^ cells/ml in Neural Stem Medium composed by Dulbecco’s modified Eagle’s medium DMEM/F12 (Gibco, Burlington, Ontario), supplemented with 1% N2 (Gibco), 2% B27 (Gibco), 20 ng/ml epidermal growth factor (EGF; R&D Systems, Minneapolis, MN), and 20 ng/ml basic fibroblast growth factor (bFGF; R&D Systems) into six-well ultra low attachment plates (Corning, Turin, Italy). Spheres formation was monitored every day and 300 μL of new medium with fresh factors were added every 3 days. After 12 days spheroids were mechanically and enzymatically dispersed with Acutase (Stem Cell technologies, Milan Italy) to single cell suspension with Acutase. The single cell suspension was used in part for secondary spheres formation assessment (plated at 1000 cells/mL) and for stem cell surface markers evaluation. For flow cytometry analyses, 150,000 cells per tube were stained with anti-human 7-AAD (BD), CD133-APC (Miltenyi Biotec), anti human ABCG2-PE (R&D Systems) and analyzed with a FACS Canto II device. Briefly, CD133 and ABCG2 expression was evaluated as the percentage of positive cells on 7-AAD negative viable cells. DAOY and ONS-76 derived-spheroids were also subjected to quantitative polymerase chain reaction (qPCR) for *Oct-4* and *Sox-2* expression. Total RNA was extracted with Triazol reagent (Life Technologies). First-strand cDNA was synthesized using the iScript Kit (BioRad) followed by amplification with iQSyberGreen kit (BioRad). The following PCR conditions were used: 50°C for 2 minutes, 95°C for 2 minutes, and 40 cycles of 95°C for 15 seconds and 60°C for 30 seconds. qPCR was performed using iQ-SYBR Green qPCR SuperMix (Biorad) on a IQ^TM5^ Multicolor qPCR thermal cycler (Biorad). The ΔΔCt method was used to estimate the fold change expression over control samples. All values were normalized to glyceraldehyde 3-phosphate dehydrogenase (*GAPDH*).

### Evaluation of 4-HPR effects on MB-derived spheroids

DAOY and ONS-76 cells were seeded at 1 x 10^4^ cells/ml, in NSC medium, into ultra low attachment six wells plates (Corning) and treated with 4-HPR at 100-250-500-1000 nM; cells were pulsed every 3 days by adding 4-HPR and 300 L of fresh medium, for 12 days. The effect of 4-HPR on sphere morphology and number was monitored with a Zeiss Microscope associated with a Nikon camera. Spheres falling in 5 random selected different fields for each well were counted. Flow cytometry analyses for the CD133 and ABCG2 surface markers were performed as described above. The ability of 4-HPR to modulate *Oct-4* and *Sox-2* expression level was also assessed by qPCR analyses as described above.

### Evaluation of 4-HPR effects on MB parental and tumor initiating cells migration and invasion

Chemoinvasion and chemotaxis assays were performed in modified Boyden chambers, as described previously [55, 56]. Briefly, viable DAOY and ONS-76 cells (5 x 10^4^), treated with 4-HPR (2.5, 5 and 10 μM) for 24 h, were washed with PBS, resuspended in serum-free medium and placed in the upper compartment of the chamber. Chemoattractant (FBS) in EMEM or RPMI, for DAOY and ONS-76 respectively, was added in the lower compartment. 10 μm pore-size polycarbonate filters were pre-coated with matrigel (1 mg/ml, BD) for the chemoinvasion assay, and with collagen IV (50 μg/ml, Sigma Aldrich) for the chemotaxis assay. After 24h (chemoinvasion) or 6h (chemotaxis) of incubation, the filters were recovered, cells on the upper surface mechanically removed and migrated or invaded cells on the lower filter surface were fixed with absolute ethanol and stained with DAPI. Cells were counted in a double-blind manner in 5 consecutive fields each with a Zeiss Microscope associated with a Nikon camera. Experiments were performed in triplicate. The same protocol was applied to evaluate whether 4-HPR treatment affected invasive ability of BTIC derived from MB spheroids.

### Immunoblotting analysis

MB cells were treated for 6 or 24 h with 4-HPR (2.5, 5, 10 μM). Lysates were prepared using Cell Lysis Buffer (Cell Signaling Technology) and protein concentrations evaluated by the DC Protein Assay (Bio-Rad, Hercules, CA, USA). Protein extracts (20 μg) were separated on 8 or 12% SDS—polyacrylamide gel electrophoresis under reducing conditions and transferred onto polyvinylidene difluoride (PVDF) membranes (Amersham, Biosciences, Otelfingen, CH, USA) that, following blocking with 5% bovine serum albumin (BSA) and 0.1% Tween-20, were then incubated with the antibodies (dilution 1:1000) directed against the following human antigens: Caspase-9, AKT, WNT3a, β-Catenin, Phospho-GSK3-β (Ser 9), Axin-1, STAT3, NF-kB, phospho-Checkpoint kinase2 (Chk2), Cyclin D1, Cyclin-dependent kinase 4 (CDK4), Nrf2, Vinculin, β-actin, GAPDH, tubulin (all from Cell Signaling Technology). The primary antibodies were diluted in Tris buffer supplemented with 5% BSA and 0.1% Tween. PVDF membranes, following rinsing in TBS 0,1% Tween-20 were then incubated with horseradish-peroxidase-conjugated anti- rabbit or anti-mouse secondary antibodies (Cell Signaling Technology; 1:5000) and reactions were visualized with the enhanced chemiluminescence kit (ECL plus, Amersham, Biosciences, Otelfingen, CH, USA).

### In vivo xenograft tumor cell growth

All the procedures involving the animals and their care were conformed to the institutional guidelines, in compliance with national and international laws and guidelines for the use of animals in biomedical research and housed in pathogen-free conditions. All the procedure applied were approved by the local animal experimentation ethics committee Comitato Etico per la Sperimentazione Animale (ID#05/13) of the University of Insubria and by the Health Ministry. CD1 nu/nu female mice (age 6–7 weeks) were obtained from Charles River Italia (Calco, Italy). Animals (5 per group) were injected intraperitoneally (IP) with 12 mg/kg 4-HPR following a prevention/intervention protocol: 2 days prior tumor cell inoculation and every 2 days throughout the course of the experiment. The injection mixture was composed of 12.5% of 4-HPR (dissolved in 100% ethanol), 12.5% of polyethoxylated castor oil vehicle (CREMOPHOR, Sigma Aldrich) and 75% of PBS. The same mixture with ethanol alone was used for the control group. On day 0, DAOY and ONS-76 cells (8 x 10^5^) mixed with 100 μL 10 mg/mL liquid Matrigel (BD) and 12 mg/Kg 4-HPR or control solution to a final volume of 300 μl, were injected subcutaneously [57] in the flank of the nude mice. Tumor size was monitored by measuring length and width with a caliper every 2 days. Mice were sacrificed 24 days after tumor cell injection, the tumors were removed and photographed. Part of the tumors were enzymatically digested with collagenase IV to obtain a single cell suspension further subjected to flow cytometry analysis for the CD133 and ABCG2 stemness marker expression as described above. Part of the tumors were included in OCT (Polyscience, Eppelheim, Germany) and immediately frozen for histological examination. The tumors were cryosectioned (5μm sections) that were stained with hematoxylin and eosin.

### Statistical analysis

Data are expressed as means ± SEM. The statistical significance between multiple data sets was determined by one-way ANOVA using Graph-Pad PRISM, tumor growth curves were determined by two-way ANOVA. FACS data were analyzed by the FACSDiva software. ImageJ software was used for WB band quantification.

## Results

### 4-HPR affects cell viability of MB cell lines

To determine the inhibitory effect of 4-HPR on cell proliferation, we treated DAOY and ONS-76 cells with increasing concentrations of 4-HPR (range 100 nM-10 μM) for 24, 48, 72 and 96 hs; cell viability was determined by MTT assay. We observed that 4-HPR was able to inhibit the proliferation of DAOY MB cell lines at 5–10 μM and ONS-76 2–10 μM in a dose-dependent and time-dependent manner (S1 Fig). These data indicate that 4-HPR was active in inhibiting the growth of ONS-76 and DAOY cells, consistent with previous reports on medulloblastoma cell lines [18].

**Fig 1:**
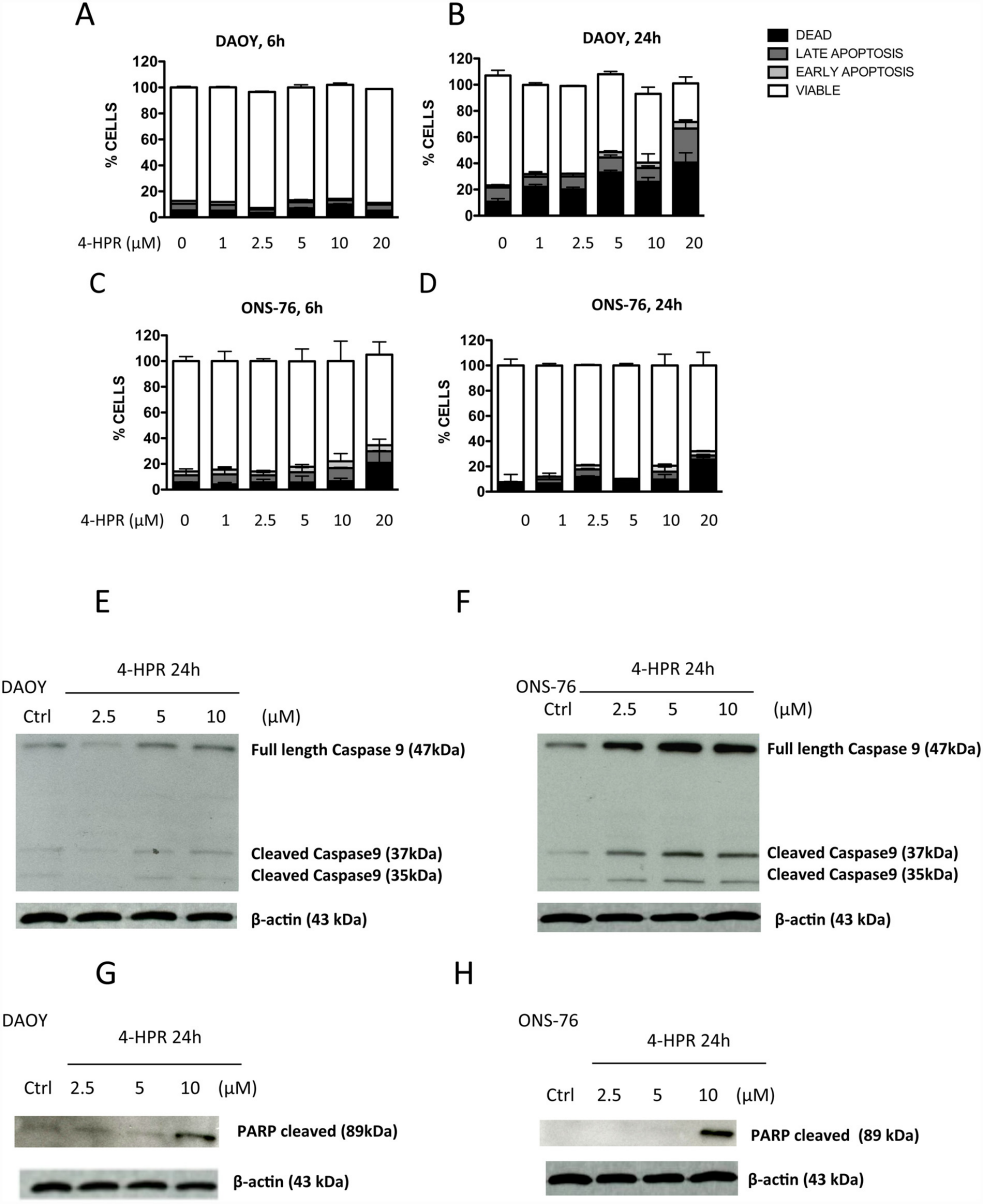
Effects of fenretinide (4-HPR) on apoptosis of MB cell lines. Flow cytometry analysis showed that treatment with 4-HPR (1–20 μM, 6–24 hours) resulted in DAOY (A, B) and ONS-76 (C, D) increased rates in apoptotic (AnnexinV^+^7-AAD^-/+^) and necrotic (7-AAD^+^) cells. Western blotting analysis (WB) for caspase-9 and PARP on cell lysate obtained from DAOY (E, G) and ONS-76 (F, H) cells treated for 24 hours with 4-HPR (2.5–10 μM) show an increase of full length/cleaved Caspase-9 and cleaved PARP-1, confirming data obtained by flow cytometry.

To evaluate whether 4-HPR triggers apoptosis in MB cells, we performed flow cytometry analyses by staining control and treated cells with Annexin V and 7-AAD. The data showed that increased concentrations of 4-HPR treatment enhanced cell death of both cell lines (Fig 1A–1D). Following 6h treatment, ONS-76 cell line seemed to be more sensitive to 4-HPR than DAOY cells. Even thougth, after 24 h treatment, a lower percentage of DAOY viable cells was observed compared with ONS-76, suggesting that longer exposition might be needed to induce apoptosis in DAOY cells (Fig 1B). Apoptosis appeared to be a key mechanism induced by 4-HPR, as confirmed by Western Blot analysis for selected mitochondrial pathway associated with caspase-9 and poly ADP-ribose polymerase (PARP) levels, on both DAOY and ONS-76 cell lines treated with 2.5, 5 and 10 μM of 4-HPR for 24h (Fig 1E–1H). 4-HPR increased the expression of full length and active caspase-9 as well as cleaved PARP expression in a dose-dependent manner after 24hs of treatment in both DAOY and ONS-76 cells (Fig 1E–1H). Enhanced levels of Caspase-9 were particularly evident in ONS-76 cells (Fig 1F). Together, these results suggested that 4-HPR triggers apoptosis in MB cells by activating a mitochondrial pathway.

### 4-HPR induces cell cycle arrest in DAOY and ONS-76 cells

**Fig 2:**
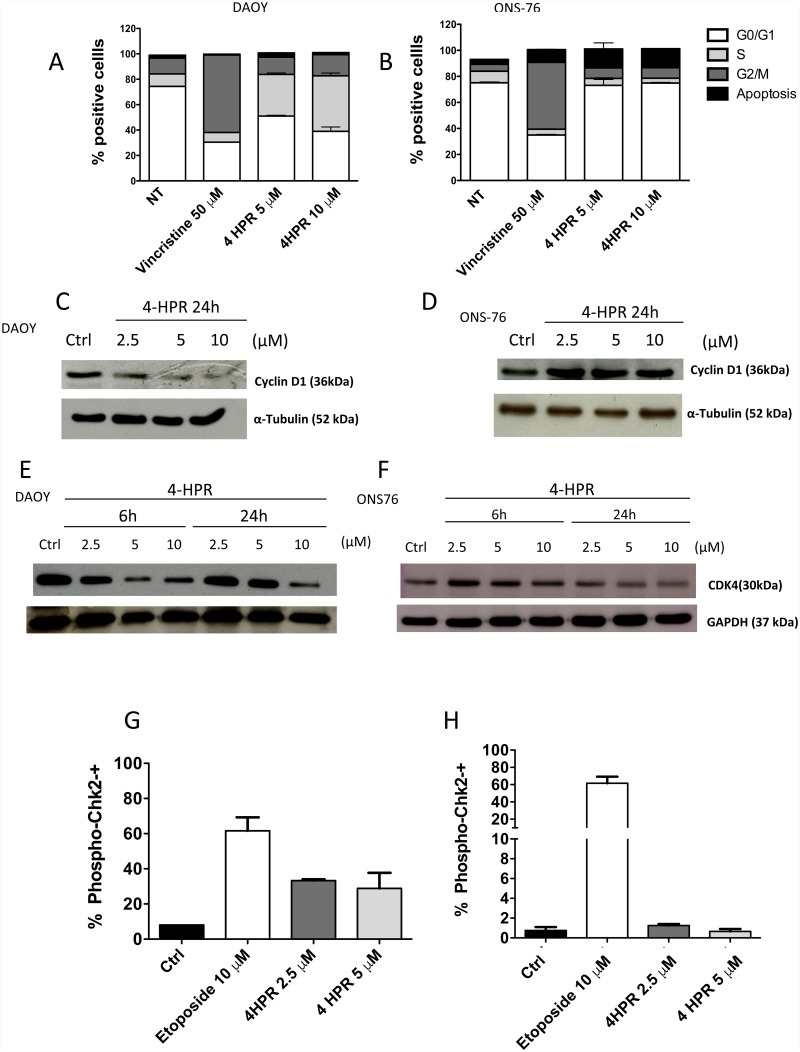
Effects of fenretinide (4-HPR) on MB cell cycle. Flow cytometry analysis (PI- staining) showed that 24-hour treatment with 4-HPR (5–10 μM) resulted in a blockage of S-phase in DAOY cells (A) while an increased rate in apoptosis was observed in ONS-76 cells (B). Western blotting analysis (WB) shows a down-regulation of Cyclin- D1 and CDK4 in 4-HPR treated DAOY. In ONS-76 cells Cyclin D1 decreased expression was not observed while CDK4 level was diminished only at the highes dose of 4-HPR (D, F). FACS analysis for phospo-Chk2 showed increased phosphorylated protein levels in 4-HPR treated DAOY confirming the cell-cycle arrest, while the same effect was not observed in ONS-76 (G, H). Vincristine (50 μM) and Etoposide (10 μM) were used as positive controls. Experiments were performed using 2 replicate for 3 repetitions.

4-HPR ability to induce cell cycle arrest on both MB cells investigated, was evaluated by flow cytometry. The data highlight that DAOY and ONS-76 showed a different behavior in response to 4-HPR treatment (Fig 2A and 2B) 4-HPR induced a dose-dependent increase in percentage of DAOY cells in the S-phase, while an enhanced apoptotic rate was observed for ONS-76 cells, confirming western blot and FACS results shown in Fig 1. Vincristine, that has already been reported to induce a cell cycle arrest in G2/M phase, was used as positive control. We also evaluated the expression of proteins involved in cell cycle regulation and checkpoints, including Cyclin D1, CDK4 and phospho-Chk2. Our results indicated that 4-HPR treatment induced down-regulation of Cyclin D1 (Fig 2C) and CDK4 (Fig 2E) expression in DAOY cells, while only 10 μM 4-HPR induced a down-regulation of CDK4 in ONS 76 cells (Fig 2D and 2F). 2.5 and 5 μM of 4-HPR were able to induce phosphorylation of Chk2 (Fig 2G and 2H), associated with an arrest of the cell cycle in S-phase.

### Effects of 4-HPR on ROS production and life survival selected pathways

Pro apoptotic effects of 4-HPR have been shown to be mediated mainly by ROS production, associated with mitochondrial membrane permeabilization and the release of cytochrome c and pro-apoptotic proteins [32, 36, 58].

**Fig 3:**
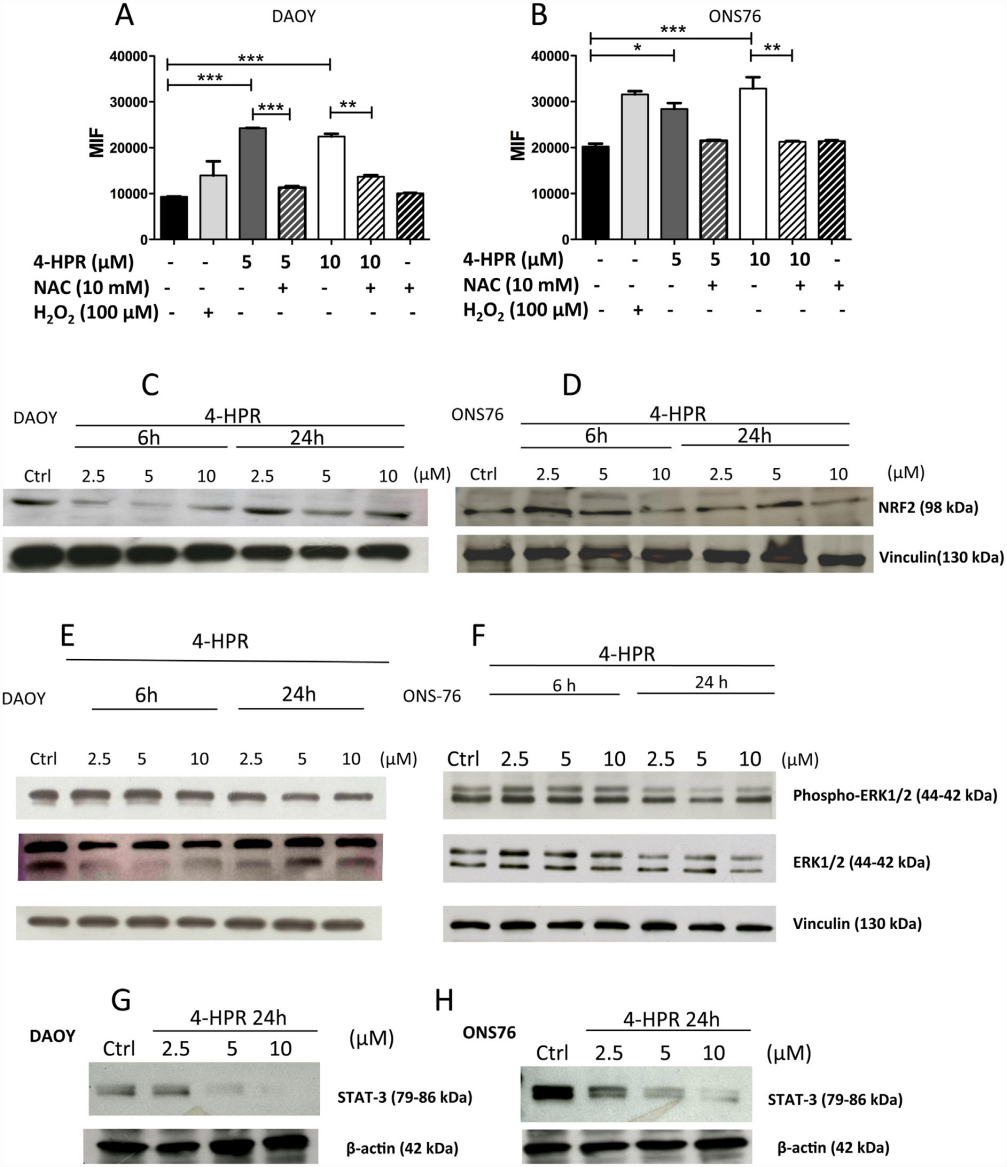
Effects of fenretinide (4-HPR) on ROS induction and MB cell survival. Flow cytometry using the H_2_DCFDA dye of 4-HPR (5–10 μM) pre-treated (2 hours) MB cells lines showed significant increased ROS production in MB cell lines (A, B), while co-treatment with the ROS scavenger NAC blocked this effect. H_2_O_2_ was used as a positive control. WB analysis for Nrf2 revealed down-regulation in 4-HPR (2.5–10 μM) treated (6–24 hours) DAOY (C) while this effect was observed in ONS-76 (D) only at the highest dose (10 μM). WB analysis for pathways involved in survival showed an overall down-regulation of pERK1/2 (E, F) and STAT3 (G, H) in 4-HPR treated (6–24 hours) DAOY and ONS-76 cells. Results are representative of 2 independent experiments and are showed as Mean ± SEM *p<0.05; **p<0.01; ***p<0.001 (one-way ANOVA).

Here we examined the effects of 4-HPR on ROS production in treated MB cells. H2O2 was used as a control radical inducer while N-acetyl cysteine (NAC), was used as a potent and widely recognize ROS scavenger. 4-HPR was able to significantly induce ROS production by MB cells (Fig 3). Treatment with 10 mM NAC decreased ROS production induced by 4-HPR and restored ROS levels to those detected in control cells (Fig 3A and 3B).

To determine the role of ROS in 4-HPR mediated cytotoxicity, we investigated the Keap1/Nrf2/ARE cytoprotective pathway, whose activation leads to the transcription of anti-oxidant enzymes involved in cellular response against oxidative stress. We observed that Nrf2 expression decreased in 6 hour 4HPR treated DAOY cells in a dose dependent manner, even at low doses, while only 10 μM 4-HPR induced a down-regulation of Nrf2 in ONS 76 (Fig 3C and 3D). These data are consistent with the resistance to 4-HPR induced apoptosis in DAOY cells and relative sensitivity to apoptosis in ONS 76 cells in response to 4-HPR treatments shown in Fig 1.

We then investigated the effects of 4-HPR on selected pathways reported to be modulated by phytochemicals in other systems [16, 31, 59, 60], such as ERK 1/2 and STAT-3 pathways. We observed a down-regulation of P-ERK 1/2 and STAT-3 in both MB cell lines, after 24h of treatment (Fig 3E–3H).

### Effects of 4-HPR on migration and the Wnt3a/β-Catenin pathway in MB cells

**Fig 4:**
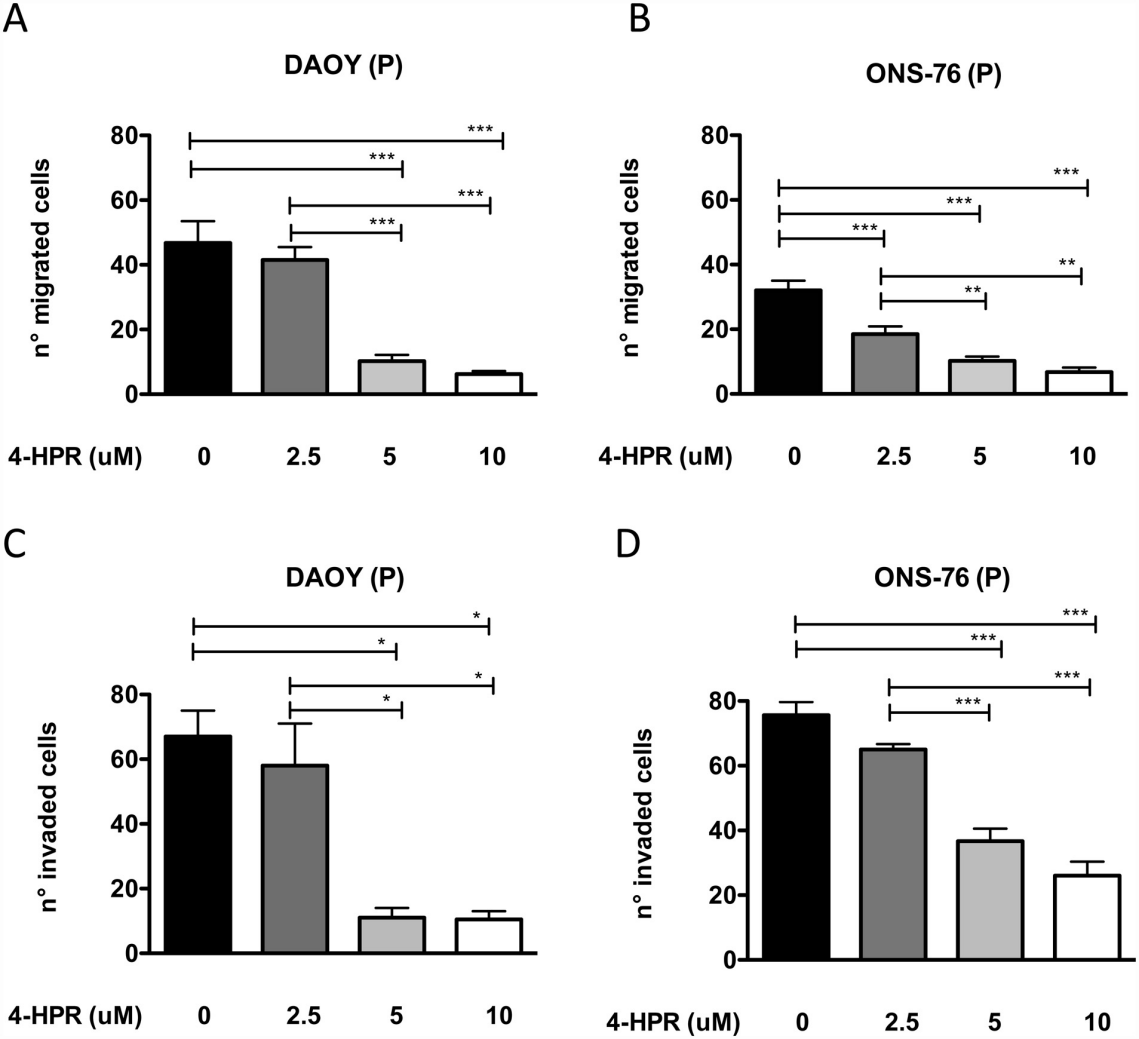
Effects of fenretinide (4-HPR) on MB cells migration and invasion. Fenretinide (4-HPR) reduces DAOY and ONS-76 cell migration (A, B) and invasion (C, D). Results are representative of 3 independent experiments and showed as Mean ± SEM *p <0.05; **p<0.001; ***p<0.001(one-way ANOVA).

**Fig 5:**
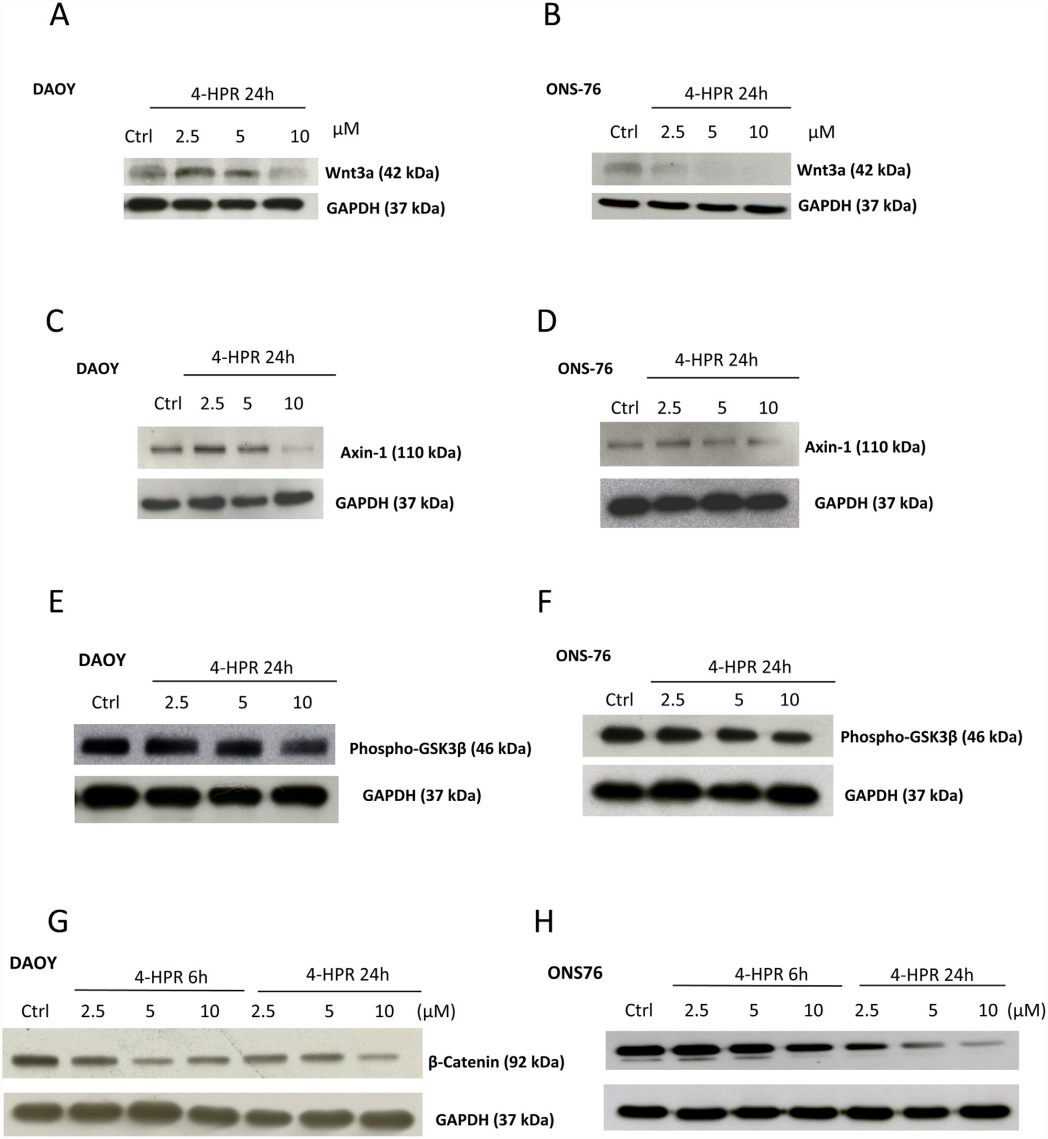
Effects of fenretinide (4-HPR) on Wnt3a/β-catenin pathway. WB analysis for proteins involved in Wnt3a/β-catenin pathway show decreased level of Wnt3a in DAOY cells (A) treated for 24 hours with 4-HPR at the highest dose while Wnt3a down-regulation started at 2.5 μM in ONS-76 cells (B). Axin-1 and pGSK3β decreased in DAOY and ONS-76 cells treated as above (C-F). Decreased levels of β-catenin was observed at 6 and 24 hours following 4-HPR treatment in DAOY and ONS-76 cells (G, H).

We also investigated the effect of 4-HPR on MB cell lines invasion and migration *in vitro*, using the chemoinvasion and chemotaxis assays, as previously described [55, 56]. 4-HPR (2.5–10 μM) inhibited both DAOY and ONS-76 migration and invasion in a dose-dependent manner (Fig 4A, 4B, 4C and 4D). Since Wnt3a/β-Catenin pathway is one of the major signaling pathways involved cancer cell migration and invasion (ref), we studied whether 4-HPR was able to interfere with the Wnt3a pathway in MB cells. 4-HPR induced a down-regulation of Wnt3a levels in both DAOY and ONS-76 cells (Fig 5A and 5B) as western blot analyses. We also investigated the effects of 4-HPR on the modulation of Axin-1, GSκ3β and β-Catenin, key downstream factors in Wnt pathway. 5 and 10 μM 4-HPR decreased the expression levels of Axin-1 (Fig 5C and 5D) and phospho-GSκ3β (Fig 5E and 5F) after 24 hours of treatment of both MB cell lines. Decreased levels of β-Catenin (Fig 5G and 5H) were also observed following 4-HPR treatment in both cells This could be correlated with a reduced invasion and migration of MB cells and to increased levels of apoptotic cell rates after 4-HPR treatment observed in Fig 4.

### Effects of 4-HPR on MB-derived Cancer Stem/Initiating Cells

**Fig 6:**
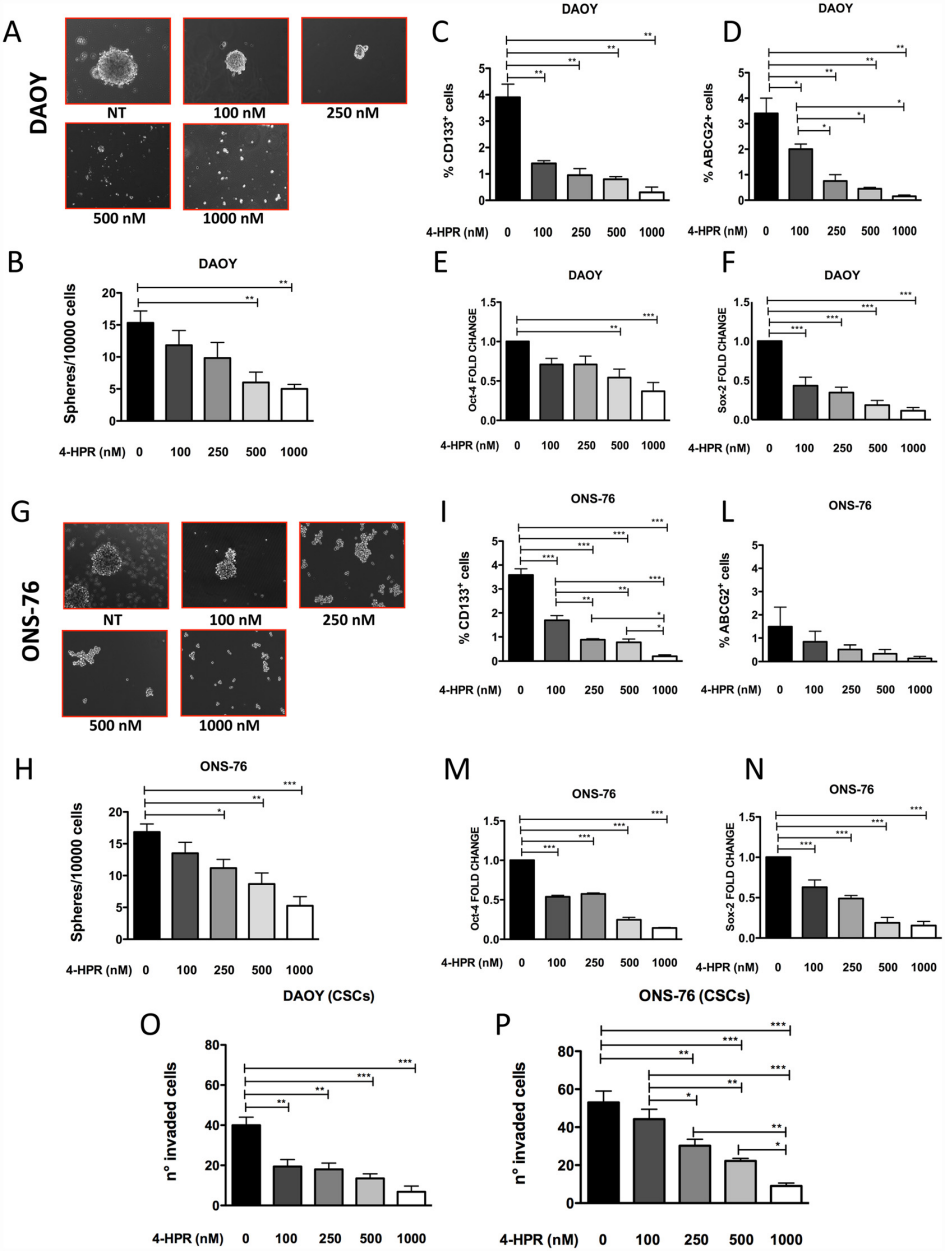
Effects of fenretinide (4-HPR) on MB brain tumor initiating cells (BTICs). Fenretinide inhibits MB spheroid formation at nanomolar concentration (100–1000 nM) in DAOY (A) and ONS-76 (B) cells cultured in stemness condition, as confirmed by sphere counts (B, H), following 4-HPR exposition. The inhibition of spheroid formation is associated by decreased levels of CD133 and ABCG2, as showed by flow cytometry (C, D; I, L) as far as down-regulation of *Oct-4* and *Sox-2* genes (E, F; M, N), as assessed by q-PCRC. Results are showed as Mean ± SEM, *p <0.05; **p<0.001; ***p<0.001. Fenretinide (4-HPR) decreases DAOY and ONS-76 BTIC invasive activity (O, P). Results are showed as Mean ± SEM, *p <0.05; **p<0.01; ***p<0.001. Results are representative of 3 independent experiments.

Effects on BTIC-like in MB cells were assessed by evaluating the ability of 4 HPR to inhibit spheroid formation. When cultured in stemness conditions, DAOY and ONS-76 cells are able to form floating spheroids typical of BTICs. These spheroids, that represent a validated and widely used method to study cancer stem cells *in vitro* [61–63], are enriched in cells with stem-like phenotype, expressing CD133, ABCG-2, *Oct-4*, *Sox-2* that are directly associated with stemness features and tumor formation *in vivo* [48, 53, 63–65]. Treatment with 4-HPR at nanomolar concentrations inhibited spheroid formation by DAOY and ONS-76 in terms of size (Fig 6A and 6G) and number (Fig 6B and 6H) in a dose dependent manner. This impaired ability to form spheroids is associated with decreased levels of CD133 and ABCG-2 (Fig 6C, 6D and 6I–6L), as assessed by flow cytometry and *Oct-4*, *Sox-2* expression (Fig 6E, 6F, 6M and 6N) determined by qPCR. 4-HPR is able to inhibit invasive abilities in DAOY and ONS-76 derived-BTICs (Fig 6O and 6P), BTICs of both DAOY and ONS-76 appear to be much more sensitive to 4-HPR than the parental cells.

### Effects of 4-HPR on MB DAOY and ONS-76 cell growth in vivo

To evaluate the ability of 4-HPR to inhibit *in vivo* tumor growth in DAOY and ONS-76 cell lines, CD1 nu/nu female mice (age 6–7 weeks) were injected intraperitoneal (IP) with 12 mg/kg 4-HPR, corresponding to the concentration range used *in vitro* (approximately 7.6 μM), 2 days prior subcutaneous tumor cell inoculation in the mice flank and pulsed every 2 days throughout the course of the experiment. Tumor growth was monitored for 24 days using a caliper according to the formula V = ½ (LxW^2^).

**Fig 7:**
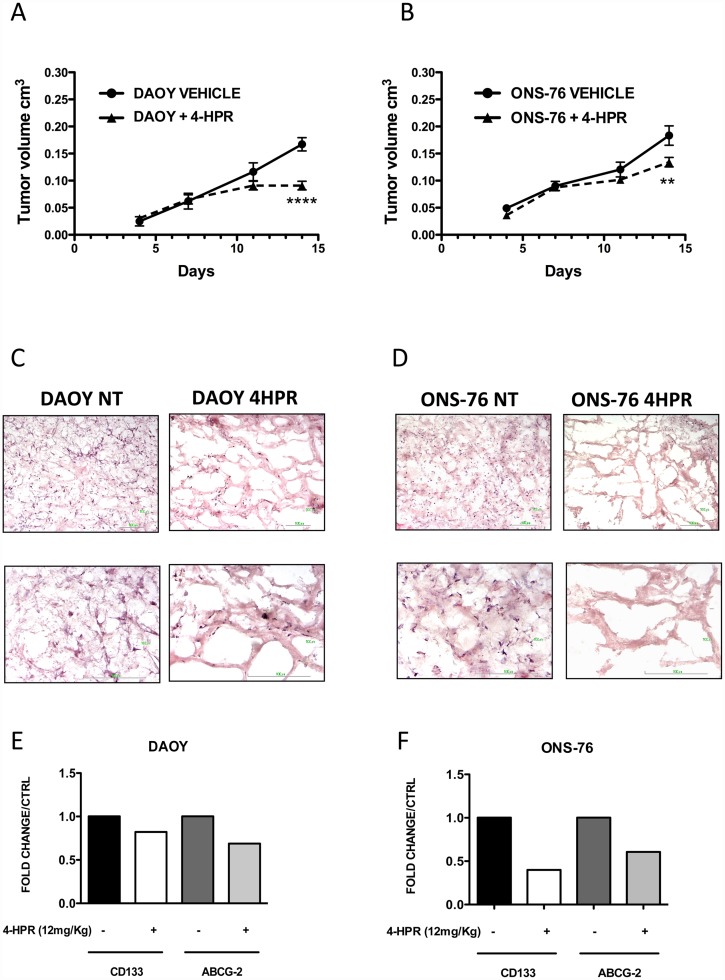
Effects of fenretinide (4-HPR) on MB tumor cell growth in vivo. Fenretinide interferes with MB tumor cell growth of subcutaneously injected cell lines (A-B). Results are shown as Mean ± SEM, *p <0.05; **p<0.001; ***p<0.001 (two-way ANOVA). Hematoxylin/eosin (5X and 20X magnification) for sections derived from DAOY and ONS-76 excised tumors shows an altered tumor tissue organization in 4-HPR-treated animals, associated with the induction of cisternae-like structures (C-D). FACS analysis for CD133 and ABCG2 expression in DAOY and ONS-76 tumors (E-F). 5 mice per group were used.

In DAOY tumors, the differences between the treated and the control mice were observed after 9 days and became highly significant from day 14 (Fig 7A). The difference for ONS-76 tumors was also significant at day 14 (Fig 7b), although these cells appeared less sensitive to 4-hpr. MB tumors formed a consistent structure while MB tumors from 4-HPR treated mice were a soft mass. Histology showed the presence of cysts, likely filled by liquid resulting in a porous-sponge like tissue (Fig 7C and 7D). Facs analysis on tumor cells pooled from tumors derived from either treated or control animals, revealed decreased number of CD133^+^ and ABCG2^+^ BTICs in tumor from 4-HPR treated animals (Fig 7E and 7F) confirming *in vivo* the ability of fenretinide to target the MB stem component shown *in vitro* (Fig 6).

## Discussion

Retinoids have been reported to play a relevant role in the regulation of cell differentiation [66], immune cell function [67], tumor growth and development [68]. Currently, one of the most promising clinically tested synthetic retinoid analogues is fenretinide (4-HPR). 4-HPR has shown biological activity against numerous cancer cell types *in vitro*, *in vivo* and in preclinical studies. 4-HPR has been reported to exhibit anti-tumor effects on breast cancer cells, through suppressing NF-KB activation and inhibiting matrix metalloproteinase-9 expression [21–23], on prostate carcinoma cells, by modulating the pro-apoptotic/anti-apoptotic protein ratio, leading to an alteration of mitochondrial membrane potential and down regulating the levels of survivin [24, 25], and myeloid leukemia [28], human pancreatic cancer cells [27].

Recently, Mittal et al demonstrated that fenretinide exhibited a relevant anti-tumor activity on endometrial cancer cells, both *in vitro* and *in vivo* [69]. 4-HPR has been reported to decrease cell viability of endometrial cells inducing apoptosis mediated by PARP and caspase-9 activation [69]. Antiangiogenic and angiopreventive properties by 4-HPR have been also reported [31, 60, 70]. 4-HPR shows potential synergy with cytotoxic drugs: the combinations 4HPR/cisplatin, 4HPR/paclitaxel and 4HPR/etoposide were more than additive compared with monotherapy in human lung cancer cells [19, 71]and the combination of fenretinide/indole-3-carbinol has increased the cytotoxic activity compared with agents administered as monotherapy by enhancing apoptosis in human breast cancer cell lines [72].

During the last decades, given the promising data obtained in pre-clinical studies, several clinical trials have been developed demonstrating that 4-HPR is a highly active and promising therapeutic agent against breast cancer [21, 29, 30, 72], lung cancer [19, 71, 73] and neuroblastoma [74, 75]. Preventive activity for secondary breast cancer by 4-HPR has also been reported [29]. Clinical trials from patients with glioma indicated that 4-HPR is able to cross the blood brain barrier (BBB), suggesting its potential for the treatment of central nervous system tumors [37, 38, 40].

Medulloblastoma (MB) is a highly aggressive pediatric tumor of the cerebellum, usually representing the most common malignancy of the cerebellum in childhood. MB accounts for 13–20% of all pediatric central nervous system tumors [1, 2]. Recently, on the basis of gene expression and immunohistochemistry analysis, four subtypes of MB have been recognized and termed as Wingless (WNT), Sonic Hedgehog (SHH), Group 3 and Group 4 [76, 77], suggesting that the molecular differences of MB groups may be relevant also for clinicians and may predict the responsiveness to treatment [9, 12]. In the brain tumor context, preliminary data from Damodar et al showed the evidence that fenretinide was able to affect human MB cell viability *in vitro*, inducing apoptosis mediated by the activation of caspase-3 and supported by the cleavage of PARP-1 [18], even if more detailed mechanisms and molecular targets have not been investigated.

Here we investigated the effects of 4-HPR on the different pathways that regulate MB proliferation, survival, migration and invasion. In this study, two different and well characterized human MB cell lines were used, DAOY cells which demonstrate properties of SHH tumors [78], and ONS-76, described as a more immature cell line [54].

According to the literature, we show that in both MB cell lines, 4-HPR treatment was able to inhibit cell proliferation, mainly due to the induction of apoptosis (Fig 1C, 1D, 1F and 1H) in ONS-76 cells, while in DAOY cells we also observed a cell cycle arrest in S phase (Figs 1A, 1B, 1E and 1G and 2A, 2C, 2E and 2G). 4HPR-induced apoptosis on MB cell lines appeared to be due to activation of the mitochondrial pathway, mediated by caspase-9 whose levels were enhanced in both MB cell lines after treatment (Fig 1E and 1F).

We also observed that 4-HPR was able to interfere with pro-survival pathways, including MAPK and STAT-3 signaling, down-regulating the expression levels of both P-ERK-1/2 and STAT-3 (Fig 3E, 3F, 3G and 3H), suggesting that the effects on caspases are not the only mechanism through which fenretinide exerts its anti-tumor activity.

Several studies demonstrated that the increase of ROS and mitochondrial alterations are involved in 4HPR-induced cell death of different brain tumor cell lines *in vitro* [33], including in MB cells [18]. According with this evidence, we observed that a substantial increase in intracellular ROS production occurred in 4HPR-treated DAOY and ONS-76 MB cell lines (Fig 3). We also found that DAOY and ONS-76 are differently able to produce ROS, following 4-HPR treatment. Indeed, it appears that the ONS-76 cell line had a higher baseline level of ROS than DAOY (Fig 3A and 3B). The antioxidant NAC is able to prevent ROS production in MB treated cell lines, consistent with previous reports indicting that L-ascorbic acid, another antioxidant, prevented cell death in MB cell lines [18] indicating that free radicals can mediate MB cell death. We also observed that 4-HPR was not only able to enhance ROS production, but it was also able to affect Nrf2 anti-oxidant pathway, down-regulating Nrf2 expression levels after 6h treatment in both MB cell lines, particularly in the DAOY cell line (Fig 3C and 3D).

The Wnt pathway and its different components play a pivotal role in several malignancies including MB [79]. Normally, in the absence of Wnt signal, GSκ3β and Axin-1 are complexed with β-Catenin and this complex favors β-Catenin phosphorylation, driving its degradation. Zhang et al have previously demonstrated that fenretinide was able to affect Wnt signaling, showing that in treated leukemia stem cells, 4-HPR suppressed β-catenin, its directly associated transcription factor *LEF1*, the Wnt pathway activators *MYCN* and *PRKCH*, and the Wnt target genes *CCND1* and *c-MYC* [80]. According to these findings, our data showed that 4-HPR affected Wnt3a signalling pathway in MB, down-regulating Axin-1 and P-GSκ3β expression (Fig 4). Reduced activation of Wnt3a pathway resulted in decreased level of β-Catenin, leading to inhibition of different genes involved in cell cycle, including cyclin D1, whose levels were down-regulated by 4-HPR treatment, blocking G1/S phase transition.

Treatment with 4-HPR resulted also in reduced invasion ability of MB cell lines, as observed in the chemotaxis and chemoinvasion assay, where 24h pre-treatment with 4-HPR 2.5, 5 and 10 μM of DAOY cells significantly reduced invasion in a dose dependent manner (Fig 6A–6C and 6B–6D). ONS-76 cells, seems to be less affected by 4-HPR. The ability of fenretinide to impair migration and invasion properties in vitro was previously demonstrated for human prostate cancer cells, where 4-HPR treatment downregulated FAK and AKT and enhanced β-catenin degradation leading to the suppression of its target genes, including cyclin D1, survivin and VEGF and decreasing invasive and migratory properties [16].

Since MB is a tumor characterized by an “embryonic” phenotype [42, 51], we evaluated whether 4-HPR was able to affect the spheroid model for MB cancer-stem-like/cancer initiating cells (BTICs). When cultured in stem-like condition [63], both DAOY and ONS-76 cells are able to form large spheres (Fig 5A, 5B, 5G and 5H). We show that 4-HPR inhibited the DAOY and ONS-76 capability to form spheroids. 4-HPR was able to decrease the expression CD133 and ABCG-2 surface antigens as well as *Oct-4* and *Sox-2* gene expression in BTICs (Fig 5C, 5D and 5I–5L) and (Fig 5E, 5F, 5M and 5N), suggesting that 4-HPR targets the BTIC tumor component (Fig 5). 4-HPR decreased also DAOY and ONS-76 BTIC invasive ability (Fig 6E and 6F). The MB BTIC population appeared to be more sensitive to 4-HPR that the parental cells for both cell lines. Similarly, fenretinide has been shown to target acute myelogenous leukemia and chronic myeloid leukemia stem cells as well as ovarian cancer stem cells *in vitro* reducing sphere formation [28, 80, 81].

4-HPR significantly reduced the growth of MB tumors *in vivo* in the prevention/intervention approach. Histological inspection of MB excised tumor from treated animals, showed alteration in the tumor mass, with induction of cisternae-like structures (Fig 7). Finally, we confirmed the ability of 4-HPR to target the BTIC component within the tumor mass, as showed by the CD133^+^ and ABCG2^+^ reduced cell percentage in the excised tumors from treated animals.

Taken together, our study demonstrates the ability of 4-HPR to interfere with MB tumor cell growth both *in vitro* and *in vivo*, acting on several molecular targets associated with cell survival and death, cell cycle, migration, invasion and stemness that we summarized in Table 1. These findings contribute to provide the basis for the development of a new approach to control MB while limiting side effects and increasing therapeutic options.

**Table 1:** Effects of 4-HPR on selected targets in DAOY and ONS-76 MB cell lines.

BIOLOGICAL PROCESS	TARGET	DAOY	ONS-76
**Apoptosis**	Casp9	up	up
	PARP-1	up	up
**Cell survival**	ERK1/2	down	down
	pStat3	up	down
**Cell cycle**	pCHK-2	up	-
	CyclinD1	down	-
**ROS**	Nrf2	down	-
**Invasion**	Wnt3a	down	down
	pGSK3β	-	down
	β-catenin	down	down
**Stemness**	CD133	down	down
	Oct-04	down	down
	Sox-2	down	down

Effects of 4-HPR on MB cells are summarized classified for biological process: apoptosis, life survival, cell cycle, ROS, invasion and stemness features. Red = up regulation, green = down regulation, yellow = not modulated).

## Supporting Information

S1 Fig Effects of fenretinide (4-HPR) on MB cell line proliferation. Fenretinide (100 nM-10 μM) inhibits DAOY and ONS-76 MB cell proliferation in a time and dose dependent manner, as shown by an MTT assay. Results are showed as Mean ± SEM, *p<0.05; **p<0.001 ***p<0.001 (two-way ANOVA). Three independent experiments, using 12 replicates were performed.
(TIF)
