# Supplementary figures and images for: Fenretinide (4-HPR) Targets Caspase-9, ERK 1/2 and the Wnt3a/β-Catenin Pathway in Medulloblastoma Cells and Medulloblastoma Cell Spheroids
Source: PLoS One. 2016 Jul 1;11(7):e0154111. doi: 10.1371/journal.pone.0154111 (PMC4930187; doi:10.1371/journal.pone.0154111)

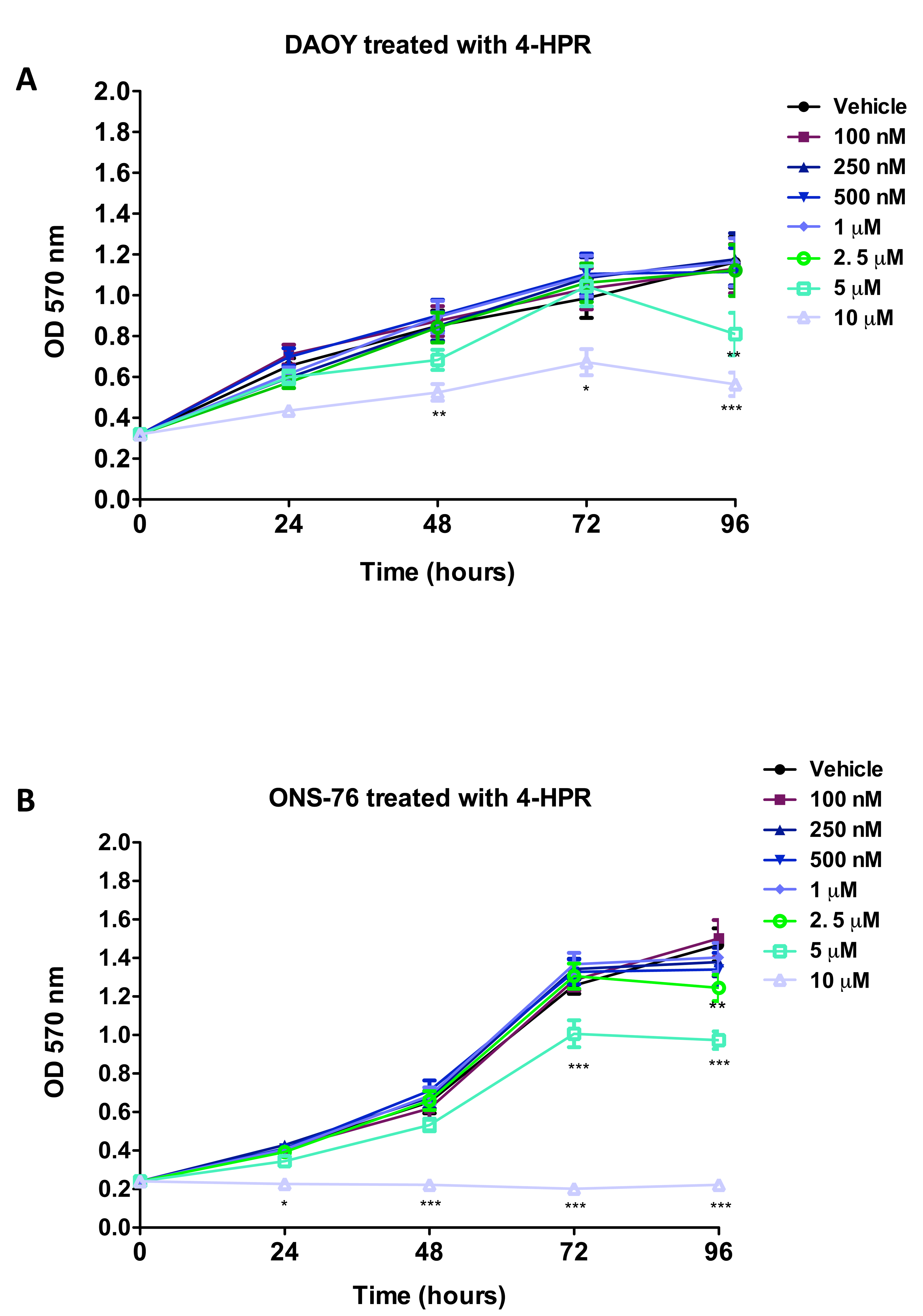

Supplement: S1 Fig — Fenretinide (100 nM-10 μM) inhibits DAOY and ONS-76 MB cell proliferation in a time and dose dependent manner, as shown by an MTT assay. Results are showed as Mean ± SEM, *p<0.05; **p<0.001 ***p<0.001 (two-way ANOVA). Three independent experiments, using 12 replicates were performed. (TIF) [file pone.0154111.s001.tif]
